# The Spatial and Temporal Dynamics of Rabies in China

**DOI:** 10.1371/journal.pntd.0001640

**Published:** 2012-05-01

**Authors:** Jinning Yu, Hao Li, Qing Tang, Simon Rayner, Na Han, Zhenyang Guo, Haizhou Liu, James Adams, Wei Fang, Xiaoyan Tao, Shumei Wang, Guodong Liang

**Affiliations:** 1 State Key Laboratory for Infectious Disease Prevention and Control, Institute for Viral Disease Control and Prevention, Chinese Center for Disease Control and Prevention, Beijing, People's Republic of China; 2 State Key Laboratory for Virology, Wuhan Institute of Virology, Chinese Academy of Sciences, Wuhan, Hubei, People's Republic of China; 3 Department Of Epidemiology and Health Statistics, School of Public Health, Shandong University, Jinan, People's Republic of China; Oswaldo Cruz Foundation, Brazil

## Abstract

**Background and Objectives:**

Recent years have seen a rapid increase in the number of rabies cases in China and an expansion in the geographic distribution of the virus. In spite of the seriousness of the outbreak and increasing number of fatalities, little is known about the phylogeography of the disease in China. In this study, we report an analysis of a set of Nucleocapsid sequences consisting of samples collected through the trial Chinese National Surveillance System as well as publicly available sequences. This sequence set represents the most comprehensive dataset from China to date, comprising 210 sequences (including 57 new samples) from 15 provinces and covering all epidemic regions. Using this dataset we investigated genetic diversity, patterns of distribution, and evolutionary history.

**Results:**

Our analysis indicates that the rabies virus in China is primarily defined by two clades that exhibit distinct population subdivision and translocation patterns and that contributed to the epidemic in different ways. The younger clade originated around 1992 and has properties that closely match the observed spread of the recent epidemic. The older clade originated around 1960 and has a dispersion pattern that suggests it represents a strain associated with a previous outbreak that remained at low levels throughout the country and reemerged in the current epidemic.

**Conclusions:**

Our findings provide new insight into factors associated with the recent epidemic and are relevant to determining an effective policy for controlling the virus.

## Author Summary

Rabies is a major problem in developing countries and responsible for more than 55,000 deaths annually. More than half of the cases occur in Asia and China has the second highest incidence of rabies after India. Human rabies cases in China decreased during the early 1990s but the virus began to re-emerge in the latter half of the decade and spread rapidly across the country with a corresponding increase in cases. To try and learn more about the epidemic, in 2006 the government implemented a trial surveillance program to sample and screen canine populations in locations where human cases were reported. In this work we selected a subset of samples (representative of the entire epidemic region) for sequencing and investigated the history and origin of the virus in China and examined the variation from a geographical perspective. Our results indicate that the epidemic is primarily composed of a younger strain with a geographical dispersion that was consistent with the recorded spread of the virus and a second older strain that corresponds to a previous epidemic. This second group exhibits a different geographical pattern, and it appears that this strain remained at low levels throughout the country and was able to re-emerge as the epidemic took hold.

## Introduction

Rabies is an enzootic disease that causes severe dysfunction to the central nervous system [1]. While cases are relatively rare in developed countries, the virus has significant impact on a global scale, with more than 55,000 deaths reported annually [2], and represents a major public health issue in many countries. More than half of these cases occur in Asia and China has the second highest incidence of rabies after India [3], [4]; in the last 60 years several rabies epidemic waves have been reported in China and improving the understanding of how these epidemics emerge can help to determine how to best reduce the likelihood of future outbreaks. Human rabies cases in China decreased during the first half of the 1990s with a low of 159 cases reported in 1996 [5], [6], [7] but subsequently the number of human rabies cases increased dramatically, with 3,302 cases reported in 2007 [5]. At the same time the geographic distribution and the range of infected hosts has also expanded [3].

There are already many published reports on the phylogenetic relationship amongst strains isolated in China that have primarily focused on sample classification and estimation of features such as date of the most recent common ancestor (TMRCA) [3], [4], [6], [7], [8], [9], [10]. In this work we have expanded on the work of previous studies by using a more complete sequence set consisting of sequences spanning a 720 nt region of the nucleocapsid gene that encompasses samples from the entire epidemic region. Additionally, we perform a more extensive analysis of the sequence set and examine the epidemic from a phylogeographic perspective.

In 2005, in order to improve rabies control and prevention, the Chinese government implemented a trial surveillance program to monitor rabies at the national level in an attempt to obtain a more comprehensive epidemiological dataset. In addition to recording statistics on human cases, the Institute for Viral Disease Control and Prevention of China CDC cooperated with the provincial CDC laboratories and began collecting samples from dog populations in regions where human rabies cases had been reported; these samples were then screened for presence of the rabies virus by both DFA and RT-PCR detection. The positive samples were then submitted for DNA sequencing and combined with a second subset of selected sequences from publicly available sequences.

Although dogs remain the major infection source, contributing 85%–95% of human cases in China [9], the number of reported incidences caused by wildlife has also increased [11], [12]. Thus, as part of this study, we also included available samples from wildlife to examine their contribution to the current epidemic. The final sequence set represents the most comprehensive dataset from China to date, representing 210 sequences (including 57 new samples) from 15 provinces and covering all epidemic regions.

In this study we use this dataset to investigate the dissemination of the virus across China as the epidemic took hold and we analyze the sequence set in terms of genetic diversity, patterns of distribution and evolutionary history.

## Materials and Methods

### Epidemiological data

Data on human rabies cases in China between 1996 and 2008 were collected from the annual reports of Chinese Center for Disease Control and Prevention (China CDC). Human rabies cases in China are defined according to clinical symptoms and subject's case history (such as a record of close contact with infected hosts via bites or scratches) and are confirmed by laboratory test where possible. Although not all patients are confirmed by laboratory test, the typical symptoms of human rabies cases are very distinct and misdiagnosis is unusual.

Cases are recorded as part of the national infectious disease reporting system set up by the Chinese government for monitoring several diseases including rabies. If a subject at a local health service center, hospital or other health institute is diagnosed with rabies virus as outlined above this institute has the responsibility of immediately reporting the case to the local CCDC who liaise with the national headquarters.

### Sample collection, detection and sequencing

Samples were collected as part of a national surveillance program. In this program, reported human cases of rabies were followed up by visits by provincial CDC laboratories to the region. Since 85%–95% of laboratory-confirmed human rabies cases in China could be associated with a dog bite [9], specimen collection focused on dog brain samples from meat markets. The markets were selected as they are the principal location where farmers in the neighborhood sell dogs they have raised, or stray and trapped dogs, and are therefore representative of the canine population in the surrounding region. The choice of provinces and municipalities where the samples were collected were determined by incidence rate of reported human rabies cases. In every province there were 9 to15 counties selected for sample collection, which were representative regions of low, middle and high incidence rates of rabies for that province. For each county, several meat markets were chosen as sample collection locations. In this way 3275 samples were collected from the brains of dogs in 7 provinces or municipalities (Guangxi, Guizhou, Anhui, Zhejiang, Jiangsu, Shandong, and Shanghai) in China between 2003 and 2008 and screened for the presence of the rabies virus [11]. Fifty-eight brain tissue samples that tested positive for the Rabies Virus by both direct immunofluorescence assay (DFA) and RT-PCR [10], [12], [13], [14] were selected for nucleotide sequencing of a 720 nt region of the nucleocapsid gene (636 nt to 1353 nt) as described previously [3]. This region was selected as it represented the most variable section of the gene. Sequence coverage was 4×. All sequences were submitted to Genbank and accession numbers are listed in Table S1.

### Phylogenetic analysis

Additional rabies sequences were downloaded from GenBank and a subset was selected based on the following criteria: (1) that the sequence spanned the 720 nt region of the N gene from 636 nt to 1353 nt; (2) the full background information (isolation time/host/location) was available. Finally, T-COFFEE was used to identify samples with the greatest nucleotide diversity. This reduced the original set of 176 published sequences to a subset of 153 sequences that provided the greatest coverage of geographical regions and host species. These sequences were isolated from dogs, cats, deer, raccoon dogs, striped field mice (*apodemus agrarius*) and ferret badgers (*Melogale moschata*) from 15 provinces that represented the majority of regions with the most serious rabies problem (85.5% of reported cases between 1996 and 2008). When combined with the newly sequenced samples they formed a final set of 210 sequences (Table S1).

Phylogenetic trees were constructed based on the 720-nt N-gene sequence (nt704–1423) using the Maximum Likelihood (ML) method implemented in the PHYML [15] and PHYLIP [16] software packages. For the PHYML and PHYML trees, the site frequencies were estimated and gamma site variation was selected with 4 categories. For PHYML, the gamma shape value was estimated from the data.

PHYML does not use an outgroup so trees were estimated with and without Australian bat rabies virus sequence ABL1996, the topologies of all the PHYLIP and PHYML trees were compared for consistency and no significant differences were observed. The HKY model was selected using MODELTEST [17] and parameter values for the HKY substitution matrix, base composition and gamma distribution of among-site rate variation were estimated. Bootstrap values were determined for 1000 replicates. To examine the distribution of branch lengths within clade I and clade II, the two major clades identified in the tree, a Java program was used to calculate all tip to tip distances for every leaf node within each clade (downloadable from http://srlab.whiov.ac.cn/wiv_bioinformatics/treeStr.html)). The non parametric Wilcoxon test implemented in R was then used to compare the two distributions.

### Analysis of spatial dynamics

To investigate the patterns of distribution and geographical structure of the rabies virus in China, isolates in the constructed ML tree were assigned a state according to the province in which they were collected and the tree was examined for discordant sample locations. In previous phylogeographic studies these events are referred to as migration events but, for consistency with common rabies terminology, we refer to them in this paper as translocation events. Translocation events were inferred using a parsimony method with DELTRAN optimization [18] on the constructed ML tree implemented in using the PAUP 4.0 [19] and MigraPhyla [20] software packages. Statistical significance was determined using an upper-tail Monte Carlo test of 10,000 trials for randomized datasets. A sparse false discovery rate (sFDR) correction was used to account for multiple comparisons between pairs. Only clade I and II had sufficient number of sequences for the analysis.

### Shared phylogenetic history and dispersion

To examine the relationship between locations the UniFrac software package was used to generate a distance matrix between all pairs of communities (i.e. provinces) based on an estimation of the fraction of the branch lengths of the tree which is unique to each community. Principal Component Analysis (PCA) was then used to examine the geographical structure of the data by transforming the matrix such that the greatest variation occurred in the initial principal components [21], [22].

### Bayesian MCMC evolutionary analysis and population demography

Evolutionary history, including evolutionary rates of populations (nucleotide substitutions per site per year), TMRCA and population growth models was inferred by using the Bayesian - Markov chain Monte Carlo (MCMC) method implemented in the BEAST software package [23], [24], [25], [26].

Demographic histories were inferred by Bayesian skyline reconstruction and statistical uncertainty was expressed by 95% confidence intervals of the Highest Posterior Density (HPD). The constant population size, exponential population growth and logistic population growth models were considered in turn and compared using Bayes Factors [27]. Chain lengths of 5×10^7^ and 4×10^7^ were used for clade I and clade II respectively to ensure estimated samples sizes >100 and topology of the final trees was compared with the PHYML and PHYLIP trees generated in the previous section; no significant differences were observed.

### Investigation of sampling bias

To investigate whether the observed phylogeographic structure was simply a consequence of sample size or sampling bias we defined a series of distance matrices according to location for difference in (i) sample size, (ii) geographical distance between locations, (iii) number of translocation events (iv) UniFrac distance (v) Net Relatedness Index (NRI) and (vi) Nearest Taxon Index (NTI). NRI provides a measure of the dispersion of a locality throughout a tree, whereas NTI is a measure of the clustering at the leaf nodes [28]. Both these quantities were calculated for both clades using our Java phylogenetic analysis package with 1000 random trees with 100 shuffles. To compare the geographic composition of clade I and clade II a two way contingency table was created according to collection date (2003–2005, 2006–2008) and location (SW, E) and a Pearson's Chi-squared test with a Yates' continuity correction was used to compare pairs.

## Results

### Phylogenetic analysis

**Figure 1:**
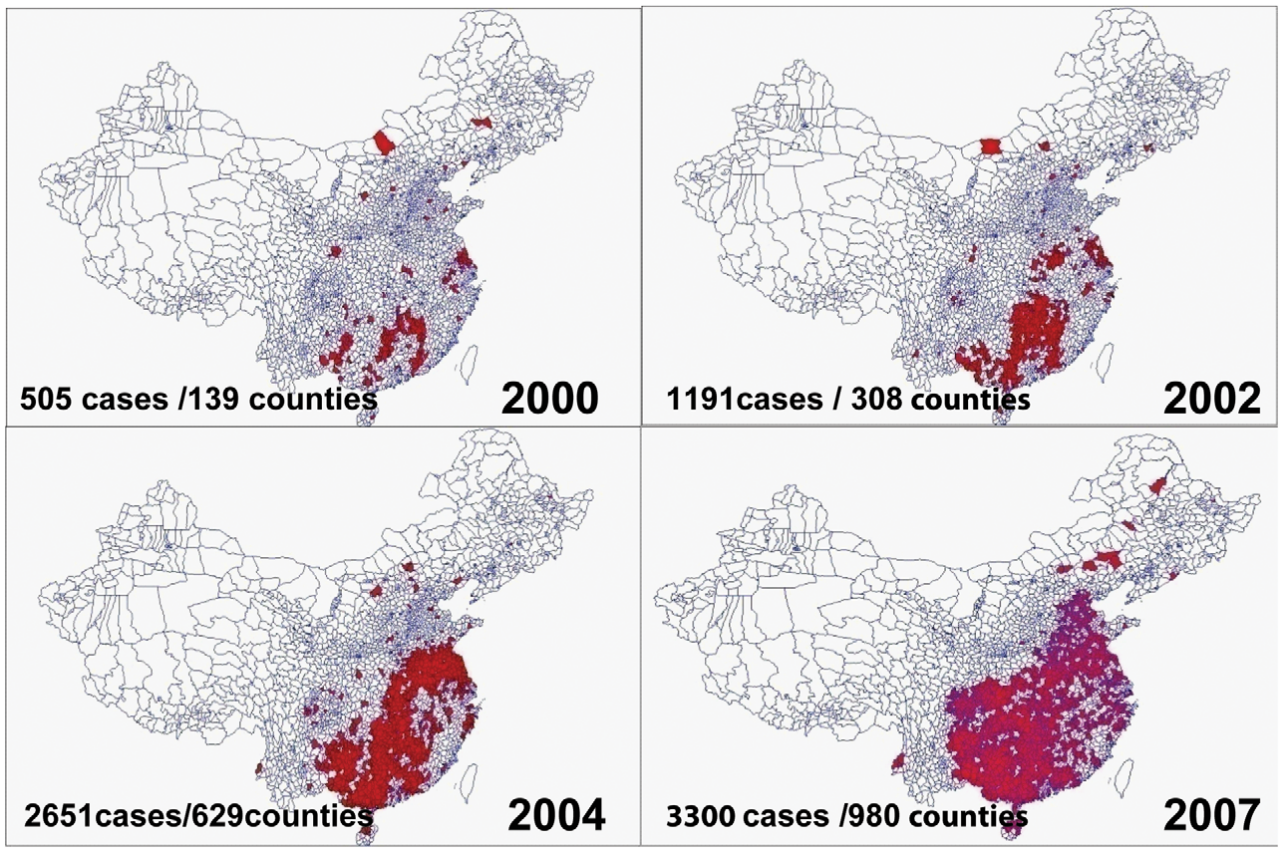
Maximum likelihood tree of 211 RABV from China partial N gene sequences generated by PHYML. Bootstrap values are indicated at the main nodes. Most sequences are contained in clade I and clade II. Underlined provinces are from the southwest, provinces with a line above the name are from the east. New isolates are marked with a blue diamond, human isolates are marked with a green cross. Clade I shows statistically significant geographic subdivision (see Table S4) according to east and southwest China with older sequences generally isolated from the southwest and sequences from the east only appearing in the younger subgroups. No such division appears in clade II. Subgroup II-A and II-C correspond to ferret badger samples (marked in red). A full list of sequences in each clade with background information is given in Table S1.

ML reconstruction of the 211 RABV partial N gene sequences collected in China divided the isolates into four major clades; clade I, clade II, clade III, and clade IV (Figure 1). Consistent with previous reports, the majority of these samples were located in clade I and II, corresponding to the Asian branch; within both of these clades further branching of the tree identified several distinct subgroups.

**Figure 2:**
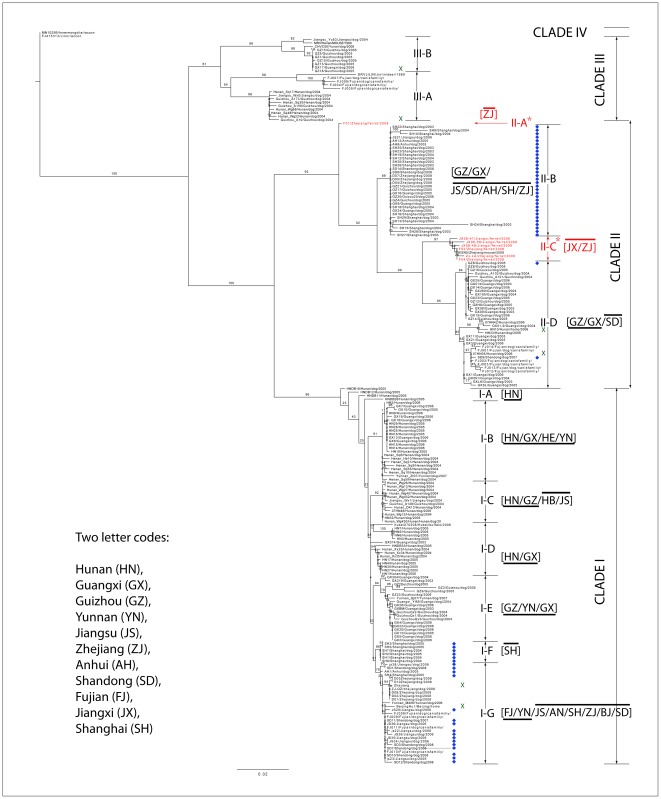
Distribution of human rabies cases in China from 2000–2007. Points represent counties where human cases were reported.

All the sequences in clade I are from dogs. Although the clade contains sequences covering all the sampled regions, the older sequences are almost exclusively from the southwest whereas the younger sequences are from the east. This is consistent with the recorded spread of the virus (i.e., number of human cases) from the southwest to the east (Figure 2). Clade II contains all wildlife samples and the Wilcoxon test indicates this clade has shorter branch lengths overall compared to clade I (Wilcoxon test W = 5002985, p-value <2.2e-16) and shows no clear geographical division, consisting of two subgroups each composed of sequences from both the southwest and the east. Another interesting feature is that sample F01, isolated from ferret badgers in Zhejiang, is placed at the earliest branch of this clade, and subgroup IIC (consisting primarily of ferret badger sequences) is placed at the top of a second large group of samples isolated from dogs.

Clade III (n = 24) corresponds to the cosmopolitan branch and represents a more general group of strains that includes isolates from dogs, rats, deer and raccoon dog and also shows no clear geographical segregation. Clade IV is confined to samples from northeastern China and forms the arctic-related branch.

### Spatial dynamic analysis

**Figure 3:**
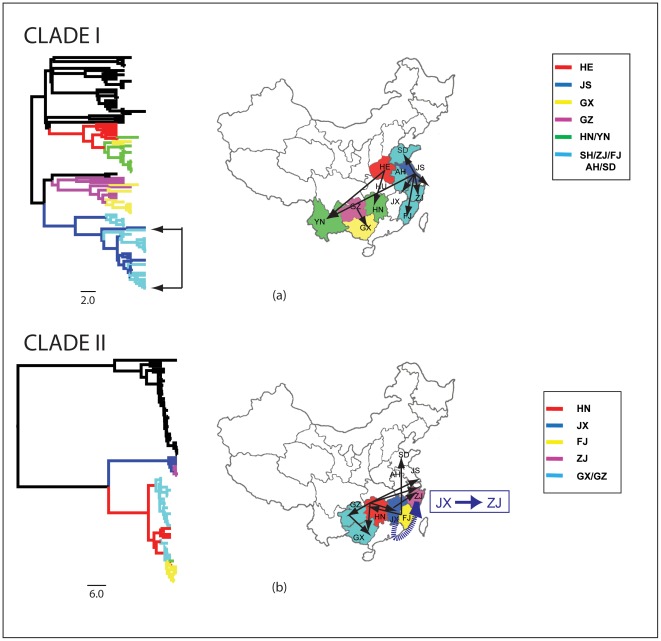
Predicted translocation events for clade I and clade II. Statistically significant (p<0.05) predicted translocation events for (a) clade I and (b) clade II. The left hand side of the figure shows the estimated BEAST tree for each clade. The branches are colored coded by location (see legend on right). For clarity, Shanghai, Zhejiang, Fujian, Anhui and Shandong are grouped together as they are bordering provinces and there are no migration events amongst them. The arrows at the bottom of the tree show the location of sequences with an ancestral sequence predicted to originate in Jiangsu (JS) province. The map on the right shows the translocation events predicted to originate from Jiangsu. These translocation events can also be seen in the marked region on the tree which, in contrast to other parts of the tree, contain multiple branches with two different colors. (b) map shows no clear centers for translocation, but a statistically significant translocation event is predicted for wildlife (ferret badger) from Jiangsu to Zhejiang province (dashed arrow on right of map).

Spatial dynamic analysis was used to identify structure in the geographic diffusion of the rabies virus in China at the provincial level. Only clade I and clade II had sufficient sequences for the analysis. All possible pair-wise comparisons were examined (110 for clade I and 90 for clade II) and a number of translocation events were identified with a high level of support (Figure 3 and Table S2). Specifically, in clade I, Jiangsu province appears to be a main source of translocation events with additional events identified as originating in Guizhou and Henan. Statistically significant events were predicted for inter-province translocation from Jiangsu province in the east to several other eastern provinces (Shandong, Fujian (p = 0), Beiing (p = 0.004), Anhui (p = 0.006) and Shanghai and Zhejiang(p = 0.05)). In the southwest of China the only events with strong support were Henan to Hunan (p = 0.01) and Yunnan (p = 0.03), and Guizhou to Yunnan (P = 0.03) and geographic subdivision is by far the strongest signal in these data. Similar to clade I, migratory centers in southwestern and eastern China were also present in the clade II samples with high support. Most notably a translocation event associated with ferret badgers clade II was predicted between Jiangxi and Zhejiang province in the east with strong support (p<0.004) (Figure 3b).

### Shared phylogenetic history and dispersion

**Figure 4:**
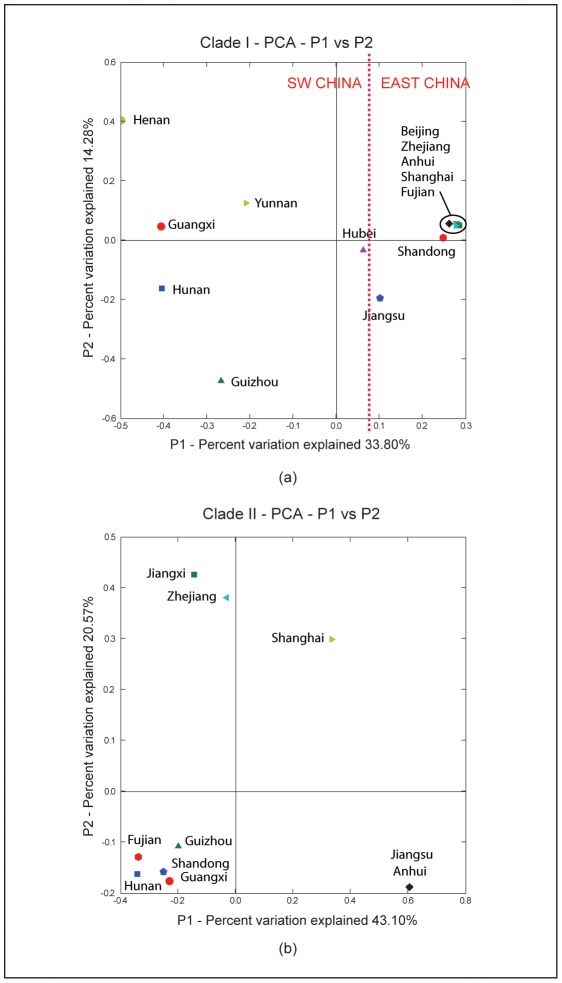
UniFrac analysis of shared evolutionary history according to location. First two principal components for UniFrac metric for (a) clade I and (b) clade II. Clade I shows a strong division between east and southwest provinces in China. The eastern provinces are closely grouped together, with the exception of Jiangsu province which is a source of multiple translocation events to southwestern provinces. Clade II shows no apparent geographical division.

UniFrac is a method that was originally developed to calculate a distance measure between bacterial communities based on the dispersion of the two communities within an estimated phylogenetic tree. The program finds taxa in the tree that contain samples from the two communities and counts the number of branches that are shared by both, or that are unique to one or the other community. To determine which provinces share similar evolutionary patterns, UniFrac was used to analyze the geographical structure of the tree by generating a distance matrix between all location pairs. PCA was then used to transform the matrix such that the greatest variation occurs in the first component, the next greatest variation in the second component and so on. The first two principal components explained 45% and 63% of the total variation for clade I and clade II respectively. The first two principal components for clade I and clade II are shown in figure 4a and b. Notably, for clade I, PC1 separates east China from southwestern China and all the eastern provinces are located to the right, with the exception of Jiangsu which is placed closer to the southwest provinces. This is consistent with the multiple translocation events that were predicted to originate from Jiangsu to provinces in the southwest. In clade II, there is no apparent geographical subdivision between southwest and east.

### Bayesian MCMC evolutionary analysis

**Figure 5:**
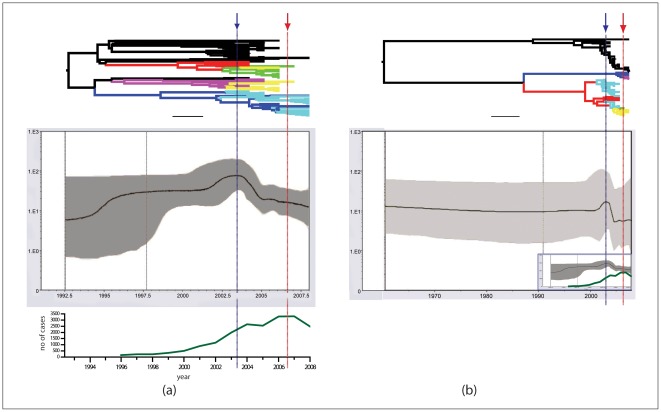
Evolutionary and transmission history of clade I and clade II. Bayesian skyline plots showing the evolutionary and transmission histories of a) clade I and b) clade II and their corresponding trees. (a) also shows the number of human rabies cases recorded by year (bottom) and (b) shows the skyline plot for clade I on the same time scale (insert bottom right). Clade I shows greater variation in genetic diversity compared to clade II. Although both clades show a drop in genetic diversity around 2003 ((a) blue arrow on left), this is not correlated to number of human cases as they were still increasing rapidly and didn't peak until 2007 (red arrow on right of (a)). However, the drop appears to coincide with the introduction of translocation events, (a) and (b) top, as at this time multiple events appear in the trees.

By using a Bayesian relaxed clock method, exponential population growth and constant population size was determined to be the most appropriate population model for clade I and clade II respectively. The evolutionary rates of each clade based on the selected population model were 1.274×10^−3^ (HPD95%: 8.3705^−4^-1.2515E^−3^)substitutions per site per year for clade I and 9.629×10^−4^ (HPD95%: 3.519^−4^-1.628E^−3^) substitutions per site per year for clade II. The corresponding TMRCA estimates for clade I, clade II and clade III were 15.5 years (about 1992; 95%HPD (10.5–20.1 years)), 48.0 years (about 1960; 95%HPD (16.1–112 years)) and 117 years (about 1891; 95%HPD (75–211 years)) respectively; because there were only two sequences for Clade IV the TMRCA was not estimated. For clade I, the virus spread from SW to E China, constantly encountering new hosts, whereas it seems that clade II was already distributed throughout the country, suggesting it was present at low levels and reemerged more gradually. Thus, the pattern of spread was very different for the two clades, and this may explain the differences in the selected population models. The Skyline plots (Figure 5) indicate clade I and clade II possess different demographic transition patterns. For clade I the genetic diversity increased rapidly from 1994 to 1996, remained relatively stable until 2001 and then underwent a second phase of rapid increase that continued until 2003. From 2004 to 2005, the genetic diversity decreased rapidly and continued a general downward trend until 2008. For clade II, the genetic diversity remained stable until 2000 when it experienced a rapid increase similar to clade I, although not as pronounced. The diversity subsequently decreased, reaching a minimum in 2005 and has remained almost constant since this time. This variation in genetic diversity in both clades is consistent with estimated times of geographic subdivision and identified translocation events. In clade I the rapid increase in genetic diversity between 1994–1996 and 2001–2003 corresponds to a significant increase in the number of branch nodes, with each sub-clade comprised of sequences from one region and little mixing between regions; this pattern was also observed in clade II between 2000 to 2003 (Figure 5a & b top). From 2004 to 2008, as the genetic diversity dropped, the number of translocation events in clade I and clade II increased; during this period the number of rabies cases continued to increase (Figure 5a bottom) so this could not be responsible for the observed drop. After this time fewer translocation events were predicted but the number of recorded rabies cases began to fall and this probably also contributed to the reduction in genetic diversity.

### Sampling bias and spatial structure

To investigate whether the observed results were due to sampling bias we generated six distance matrices based on differences between the locations and performed a pairwise Mantel test to test for correspondence (Table S3 information for results). NRI and NTI are measures of the dispersion of a location throughout the tree and for both clades there was no correlation between the number of samples and these quantities or for the number of samples and the observed translocation events.

As a further test of whether clade I and clade II possess distinct geographical structures we formed a two-way contingency table for the sample data based on sample location (SW or E) and sample date (2003–2005 & 2006–2008) and performed a chi-squared test on the sample data within and between clades (Table S4). The results indicate that for 2003 to 2005 the geographical subdivision for clade I was distinct from clade II (p = 0.027) but for 2006 to 2008 there was no difference.

## Discussion

We have performed the most comprehensive study to date of the spatiotemporal dynamics of the rabies virus in China. While previous studies of the current rabies epidemic in China have focused on the phylogenetic relationship amongst canine rabies isolates, we also attempted to investigate the possible role of wildlife.

Our identification of two major clades, clade I and II, is consistent with results from previous studies [3], [8], however this report is the first detailed investigation of the properties of these clades and the first demonstration of their distinct characteristics. Clade I is the younger of the two clades and shows geographical structure in terms of translocation events and sample dispersal. Conversely, clade II showed no clear geographical structure, consistent with its older estimation of TMRCA.

One commonly voiced concern is that the observed increase in the number of cases in China might simply be attributed to an improved surveillance program and misdiagnosis of rabies cases [29], [30]. This is unlikely for a number of reasons. Firstly, rabies surveillance data in China also includes background information for each incident and 85%–95% of reported cases can be associated with a dog bite [9]. The distinct late-stage symptoms of rabies together with the history of a dog bite means that, although misdiagnosis of other viral encephalitides remains a possibility, it is unlikely [29]. Additionally, the epidemiology of current rabies cases is not consistent with observed encephalitis outbreaks. Furthermore, although the majority of reported human cases are not verified experimentally, in the cases where it has been possible to perform laboratory diagnosis, there is a very strong correlation between clinical diagnosis and experimental verification [31], [32], [33] (and unpublished data). Secondly, there is a misconception that the surveillance program that was introduced in China in recent years represents the first effort to implement a comprehensive surveillance program in the country. What the China National Statutory Notifiable Communicable Disease Reporting System actually represents is the first attempt to coordinate efficient and automated collection of a more extensive surveillance dataset at the national level. Prior to this, data on human rabies cases was still collected at the local level, but was collated manually at the national level. The continuity between the reporting systems is supported by data from earlier periods which shows that even during periods of social unrest, details of rabies cases were recorded and the data shows clear evidence of previous epidemics [9].

What is still uncertain is the degree to which rabies is present in canines and wildlife. Currently, there is no national or local surveillance system for monitoring dog and wildlife rabies and previous estimates are based on case reports which are inconsistent and clearly underestimate the incidence in these populations. The sampling across 15 provinces in this study, although limited, is informative. Of the 3275 samples collected, 58 tested positive for the virus, corresponding to 2.8% of the dataset. As the goal of the surveillance program was to collect dog brain samples from areas where human rabies cases had been reported, this does not necessarily reflect the situation at the national level. Nevertheless, this percentage is consistent with earlier studies in China [10], [28] as well as studies in other countries experiencing rabies epidemics where larger numbers of samples were also collected from dog populations in regions where human cases had been reported and which were subsequently tested in the laboratory. For example, Vietnam reported 2 positives out of 72 (2.8%) canine samples in the former province of Hà Tây, and 5 positives out of 53 (9.4%) samples in Hô Chí Minh city [29]; Guatemala reported 25%–30% positive in dog populations sampled in the town of Todos Santos [30]; and in Bali in 2010, 144 out of 3,300 (4.8%) tested samples were positive which decreased to 67 out of 2311 (2.8%) in 2011 after the effects of widespread vaccination [31]. As more dog samples are collected as part of the National Surveillance Program it will be possible to combine these data to obtain more accurate estimates of the prevalence of rabies in the dog population in China.

Our results indicate the growth of clade I coincided with the spread of the epidemic, whereas clade II was already present throughout the sampled regions at the earliest stages. This suggests that clade II is from an earlier outbreak and existed at low levels throughout the country. This is also consistent with the earlier TMRCA for this clade and the difference in the distributions of branch lengths for the two clades.

Our results reveal the existence of both geographic dispersal and translocation events, and statistical tests indicate that it is improbable that the events are a consequence of sampling bias. Given the relatively small number of identified translocation events, it appears that geographic dispersal plays the major role in the spread of the virus. This is also supported by the observation that the branch order in the tree coincides with epidemiology data that shows that the neighboring provinces of Hunan, Guangxi and Guizhou experienced rabies outbreaks sequentially. In southwest China, Hunan seems to serve as a major source of geographic dispersal as these sequences are widely distributed among the southwestern sub-clades. The identification of translocation hotspots for clade I suggests that this mechanism also aids dissemination of the virus, although the reason why Jiangsu should act as a major translocation source is unclear. Also, because there were already cases reported in all the translocation regions, it is difficult to be certain how much translocation contributed to the epidemic. As more samples become available through the national surveillance program, it will be possible to further investigate these factors.

We also investigated the relevance of wildlife in the spread of the virus and there were a number of curious results from our study. Firstly, our phylogenetic analyses placed ferret badger sequences at the top of two distinct sub-clades of samples isolated from dogs. If the rabies in wildlife was a consequence of spillover from dogs, then we would expect to find the wildlife isolates mixed in with the dog samples. This hasn't been reported in previous studies which have either focused on dogs and only contained one or two wildlife samples [3], [4], [8], or contained more wildlife sequences but focused primarily on the epidemiology rather than the phylogenetic relationships between the strains [34]. Secondly, our study found the first evidence of a translocation event in wildlife in China. Although this is an isolated finding, the translocation event has high bootstrap support.

Previous studies have investigated rabies in ferret badgers in southeast China. While the number of isolated samples is small [34], [35], surveillance data indicates the habitat of the species extends across the entire region in which rabies events have been observed [36]. Also, a recent study reported that apparently healthy ferret badgers in Zhejiang province region had high levels of seroconversion, although this is not consistent with reports of rabies in ferret badgers in this region [34].

There are insufficient samples to draw any definitive conclusions as to whether wildlife plays a significant role in the spread of rabies in China, but our results are nevertheless interesting and further studies would be worthwhile. However, given the size of rural China, obtaining sufficient positive samples remains a formidable challenge.

It is worth noting that the current epidemic and associated increase in human cases was coincident with many social changes in the country that facilitated the spread of the disease. Firstly, vaccination represents the most effective approach to controlling rabies [37], [38] and vaccine production was previously restricted to government run laboratories. However, with the introduction of economic reforms, several private companies also began production of vaccines, some of which failed to meet national standards. This had a major negative impact on vaccination efforts in the country [39], [40]. Secondly, relaxation of control on dog ownership led to a rapid rise in dog population that was largely uncontrolled in the countryside [41]. This increase in dog population was further exacerbated by the economic reform that lead to creation of businesses in rural areas for selling dog meat, resulting in concentrated populations of unvaccinated animals [42], [43]. In order to develop an effective vaccination program, it would be worthwhile to investigate how these factors might impact the cost and efficiency of such a program. Thirdly, prior to economic reform, public healthcare ensured post-exposure prophylaxis was generally available, but with the advent of private healthcare, costs became prohibitively high for many people in rural areas [42]. While this isn't connected with the spread of rabies, it did have a major impact on the number of human cases.

This analysis on population dynamics and patterns of distribution and differentiation of the virus may help the development of a program for the prevention and control of rabies in China. Specifically, the identification of translocation hotspots suggests that these regions should be given priority in order to reduce the likelihood of reintroducing the virus into vaccinated areas. Additionally, as our results indicate that clade II is evidence of a previous epidemic, this means that the virus had maintained low levels throughout the country for an extended period and was able to rapidly reemerge when suitable conditions prevailed. The presence of these two distinct components in the epidemic needs to be taken into consideration when attempting to implement WHO recommendations [2] in regard to vaccination control programs.

## Supporting Information

Table S1 Background information of rabies sequences used in this study. Sequences are grouped according to their assigned clade in the tree shown in Figure 1. Newly sequenced strains are marked with a “+” in the right most column.
(DOC)

Table S2 Details of MigraPhyla analysis to detect significant translocation events for (a) clade I and (b) clade 2 amongst the Chinese provinces from which samples were collected in this study. Top table shows number of translocation events predicted between pairs of provinces, lower table show statistical support for the events with P<0.05.
(DOC)

Table S3 Pairwise Mantel test results for correspondence using Spearman correlation ranks amongst six different distance matrices for (a) clade I and (b) clade II. *noSamples*: number of samples in the test set, *geoDists*: geographic distances between the central point of the provinces, *UniFracPC1*: distances between the provinces calculated from the first principal component, *Migration*: Number of migration events between pairs of provinces, *NRI*: Net Relatedness Index, *NTI*: Nearest Taxon Index.
(DOC)

Table S4 Details of analysis of geographical composition of Clade I and Clade II.
(DOC)
