# Supplementary material for: The Spatial and Temporal Dynamics of Rabies in China
Source: PLoS Negl Trop Dis. 2012 May 1;6(5):e1640. doi: 10.1371/journal.pntd.0001640 (PMC3341336; doi:10.1371/journal.pntd.0001640)
Supplement: Table S1 — Background information of rabies sequences used in this study. Sequences are grouped according to their assigned clade in the tree shown in Figure 1. Newly sequenced strains are marked with a “+” in the right most column. (DOC) [file pntd.0001640.s001.doc]

**Table S1：**Background information of rabies sequences used in this study. Sequences are grouped according to their assigned clade in the tree shown in figure 1. New isolates are marked with a “+” in the right most column.

| **Clade** | **Strain** | **Host** | **Origin** | **Year** | **Genbank**  **Accession no** |  |
| --- | --- | --- | --- | --- | --- | --- |
|  |  |  |  |  |  |  |
| **Clade I** |  |  |  |  |  |  |
|  |  |  |  |  |  |  |
| **IA** | HNDB28 | Dog | Hunan | 2005 | EU008922 |  |
|  | HNDB18 | Dog | Hunan | 2005 | EU008921 |  |
|  | HNDB12 | Dog | Hunan | 2005 | EU008920 |  |
|  | HNDB11 | Dog | Hunan | 2005 | EU008919 |  |
| **IB** | CHN0525D(HN25) | Dog | Hunan | 2005 | EF990613 |  |
|  | CGX0618D(GX18) | Dog | Guangxi | 2006 | EF990592 |  |
|  | CHN0615D(HN15) | Dog | Hunan | 2006 | EF990609 |  |
|  | CGX0619D(GX19) | Dog | Guangxi | 2006 | EF990593 |  |
|  | CGX0606D(GX6) | Dog | Guangxi | 2006 | EF990583 |  |
|  | CHN0529D(HN29) | Dog | Hunan | 2005 | EF990616 |  |
|  | CHN0609D(HN9) | Dog | Hunan | 2006 | EF990607 |  |
|  | CGX0510D(GX10) | Dog | Guangxi | 2005 | EF990585 |  |
|  | CGX0607D(GX7) | Dog | Guangxi | 2006 | EF990584 |  |
|  | CGX0612D(GX12) | Dog | Guangxi | 2006 | EF990587 |  |
|  | CHN0519D(HN19) | Dog | Hunan | 2005 | EF990612 |  |
|  | CHN0516D(HN16) | Dog | Hunan | 2005 | EF990610 |  |
|  | CHN0614D(HN14) | Dog | Hunan | 2006 | EF990608 |  |
|  | CHN0528D(HN28) | Dog | Hunan | 2005 | EF990615 |  |
|  | CHN0602D(HN2) | Dog | Hunan | 2006 | EF990601 |  |
|  | Yunnan_Zt07 | Dog | Yunnan | 2007 | EU275244 |  |
|  | Henan_Sq35 | Dog | Henan | 2004 | DQ666304 |  |
|  | Henan_Sq21 | Dog | Henan | 2004 | DQ666302 |  |
|  | Henan_Sq9 | Dog | Henan | 2004 | DQ666299 |  |
|  | Henan_Sq10 | Dog | Henan | 2004 | DQ666300 |  |
|  | Henan_Sq59 | Dog | Henan | 2004 | DQ666306 |  |
|  | Henan_Hb10 | Dog | Henan | 2004 | DQ666297 |  |
|  | Henan_Sq6 | Dog | Henan | 2004 | DQ666298 |  |
| **IC** | Hunan_Wg26 | Dog | Hunan | 2004 | DQ666311 |  |
|  | Hunan_Wg13 | Dog | Hunan | 2004 | DQ666309 |  |
|  | Hunan_Wg27 | Dog | Hunan | 2004 | DQ666312 |  |
|  | Hunan_Wg407 | Dog | Hunan | 2004 | DQ666314 |  |
|  | Hunan_Wg432 | Dog | Hunan | 2004 | DQ666316 |  |
|  | Jiangsu_Wx1 | Dog | Jiangsu | 2004 | DQ666321 |  |
|  | Guizhou_A148 | Dog | Guizhou | 2004 | DQ666291 |  |
|  | Hunan_DK13 | Dog | Hunan | 2004 | DQ666307 |  |
|  | CHN0701D(07HN48) | Dog | Hunan | 2007 | EF990623 |  |
|  | Hunan_Wg12 | Dog | Hunan | 2004 | DQ666308 |  |
|  | CHN0532D(HN32) | Dog | Hunan | 2005 | EF990618 |  |
|  | Hunan_Wg430 | Dog | Hunan | 2004 | DQ666315 |  |
| **ID** | hubei070308 | Buffalo | Hubei | 2004 | EF611081 |  |
|  | CHN0505D(HN5) | Dog | Hunan | 2005 | EF990604 |  |
|  | CHN0507D(HN7) | Dog | Hunan | 2005 | EF990606 |  |
|  | CHN0606D(HN6) | Dog | Hunan | 2005 | EF990605 |  |
|  | CHN0503D(HN3) | Dog | Hunan | 2005 | EF990602 |  |
|  | GX074 | Dog | Guangxi | 2003 | DQ866107 |  |
|  | HNDB33 | Dog | Hunan | 2005 | EU008923 |  |
|  | Hunan_Xx34 | Dog | Hunan | 2004 | DQ666318 |  |
|  | Hunan_Xx33 | Dog | Hunan | 2004 | DQ666317 |  |
|  | Hunan_Xx35 | Dog | Hunan | 2004 | DQ666319 |  |
|  | CHN0527D(HN27) | Dog | Hunan | 2005 | EF990614 |  |
|  | CHN0530D(HN30) | Dog | Hunan | 2005 | EF990617 |  |
|  | CHN0601D(HN1) | Dog | Hunan | 2006 | EF990600 |  |
|  | CHN0504D(HN4) | Dog | Hunan | 2005 | EF990603 |  |
|  | CHN0517D(HN17) | Dog | Hunan | 2005 | EF990611 |  |
|  | GX219 | Dog | Guangxi | 2003 | DQ866113 |  |
|  | GX304 | Dog | Guangxi | 2004 | DQ866117 |  |
|  | CGZ0623D(GZ23) | Dog | Guizhou | 2006 | EF990577 |  |
|  | CGZ0503D(GZ3) | Dog | Guizhou | 2005 | EF990566 |  |
|  | CGZ0509D(GZ9) | Dog | Guizhou | 2005 | EF990569 |  |
|  | CGZ0502D(GZ2) | Dog | Guizhou | 2005 | EF990565 |  |
|  | Yunnan_Qj07 | Dog | Yunnan | 2007 | EU275245 |  |
|  | Guangxi_Yl66 | Dog | Guangxi | 2004 | DQ666287 |  |
|  | CGX0626D(GX26) | Dog | Guangxi | 2006 | EF990599 |  |
|  | GXBM | Dog | Guangxi | 2003 | DQ866115 |  |
|  | Guizhou_Qx5 | Dog | Guizhou | 2004 | DQ666296 |  |
|  | Guizhou_Qx1 | Dog | Guizhou | 2004 | DQ666294 |  |
|  | Guizhou_Qx2 | Dog | Guizhou | 2004 | DQ666295 |  |
|  | CGX0622D(GX22) | Dog | Guangxi | 2006 | EF990596 |  |
|  | CGX0604D(GX4) | Dog | Guangxi | 2006 | EF990581 |  |
|  | CGX0513D(GX13) | Dog | Guangxi | 2005 | EF990588 |  |
|  | CGX0620D(GX20) | Dog | Guangxi | 2006 | EF990594 |  |
|  | CGX0602D(GX2) | Dog | Guangxi | 2006 | EF990579 |  |
|  | CGX0605D(GX5) | Dog | Guangxi | 2006 | EF990582 |  |
| **IF** | CSH0501D(SH1) | Dog | Shanghai | 2005 | HM486382 | **+** |
|  | CSH0502D(SH2) | Dog | Shanghai | 2005 | HM486383 | **+** |
|  | CSH0503D(SH3) | Dog | Shanghai | 2005 | HM486384 | **+** |
|  | CSH0505D(SH5) | Dog | Shanghai | 2005 | HM486386 | **+** |
|  | CSH0407D(SH7) | Dog | Shanghai | 2004 | HM486387 | **+** |
|  | CSH0408D(SH8) | Dog | Shanghai | 2004 | HM486388 | **+** |
| **IG** | CJS0538D(JS38) | Dog | Jiangsu | 2005 | HM486353 | **+** |
|  | CSD0801D(SD1) | Dog | Shandong | 2008 | HM486376 | **+** |
|  | CAH0501D(AH1) | Dog | Anhui | 2005 | HM486360 | **+** |
|  | CJS0629D(JS29) | Dog | Jiangsu | 2006 | HM486351 | **+** |
|  | CJS0635D(JS35) | Dog | Jiangsu | 2006 | HM486349 | **+** |
|  | CJS0622D(JS22) | Dog | Jiangsu | 2006 | HM486350 | **+** |
|  | CJS0639D(JS39) | Dog | Jiangsu | 2006 | HM486354 | **+** |
|  | CJS0523D(JS23) | Dog | Jiangsu | 2005 | HM486357 | **+** |
|  | FJ008 | Dog | Fujian | 2008 | FJ866835 |  |
|  | CJS0636D(JS36) | Dog | Jiangsu | 2006 | HM486352 | **+** |
|  | CSD0807D(SD7) | Dog | Shandong | 2008 | HM748955 | **+** |
|  | BeijingHu1 | Human | Beijing | 2007 | EU700031 |  |
|  | FJ010 | Dog | Fujian | 2008 | FJ866827 |  |
|  | CSD0812D(SD12) | Dog | Shandong | 2008 | HM748957 | **+** |
|  | FJ011 | Dog | Fujian | 2008 | FJ866828 |  |
|  | CJS0634D(JS34) | Dog | Jiangsu | 2006 | HM486363 | **+** |
|  | CSD0803D(SD3) | Dog | Shandong | 2008 | HM486377 | **+** |
|  | CSD0711D(SD11) | Dog | Shandong | 2007 | HM486378 | **+** |
|  | FJ009 | Dog | Fujian | 2008 | FJ866836 |  |
|  | CSD0810D(SD10) | Dog | Shandong | 2008 | HM748956 | **+** |
|  | Yunnan_Md06 | Dog | Yunnan | 2006 | EU095330 |  |
|  | CSH0504D(SH4) | Dog | Shanghai | 2005 | HM486385 | **+** |
|  | ZJ-QZ | Dog | Zhejiang | 2008 | FJ719760 |  |
|  | ZhejiangWz1(H) | Human | Zhejiang | 2008 | EU700032 |  |
|  | D03 | Dog | Zhejiang | 2008 | FJ032315 |  |
|  | D10 | Dog | Zhejiang | 2008 | FJ032317 |  |
|  | D02 | Dog | Zhejiang | 2008 | FJ712194 |  |
|  | D01 | Dog | Zhejiang | 2008 | FJ712193 |  |
|  | D08 | Dog | Zhejiang | 2008 | FJ032316 |  |
|  |  |  |  |  |  |  |
| **Clade II** |  |  |  |  |  |  |
| **IIA** | F01 | Ferret badger | Zhejiang | 2008 | FJ032318 |  |
| **IIB** | CAH0512D(AH12) | Dog | Anhui | 2005 | HM486362 | **+** |
|  | CSH0412D(SH12) | Dog | Shanghai | 2004 | HM486355 | **+** |
|  | CSH0327D(SH27) | Dog | Shanghai | 2003 | HM486397 | **+** |
|  | CSH0414D(SH14) | Dog | Shanghai | 2004 | HM748954 | **+** |
|  | CZJ0807D(D07) | Dog | Zhejiang | 2008 | HM486375 | **+** |
|  | CSH0409D(SH9) | Dog | Shanghai | 2004 | HM486389 | **+** |
|  | CSH0328D(SH28) | Dog | Shanghai | 2003 | HM486398 | **+** |
|  | CSH0324D(SH24) | Dog | Shanghai | 2003 | HM486358 | **+** |
|  | CSH0415D(SH15) | Dog | Shanghai | 2004 | HM486392 | **+** |
|  | CGX0516D(GX16) | Dog | Guangxi | 2005 | HM486370 | **+** |
|  | CSH0419D(SH19) | Dog | Shanghai | 2004 | HM486356 | **+** |
|  | CZJ0805D(D05) | Dog | Zhejiang | 2008 | HM486374 | **+** |
|  | CSH0418D(SH18) | Dog | Shanghai | 2004 | HM486394 | **+** |
|  | CGZ0621D(GZ21) | Dog | Guizhou | 2006 | HM486372 | **+** |
|  | CSH0416D(SH16) | Dog | Shanghai | 2004 | HM486393 | **+** |
|  | CSH0326D(SH26) | Dog | Shanghai | 2003 | HM486396 | **+** |
|  | CJS0621D(JS21) | Dog | Jiangsu | 2006 | HM486364 | **+** |
|  | CSD0614D(SD14) | Dog | Shandong | 2006 | HM486380 | **+** |
|  | CSH0325D(SH25) | Dog | Shanghai | 2003 | HM486359 | **+** |
|  | CGZ0620D(GZ20) | Dog | Guizhou | 2006 | HM486369 | **+** |
|  | CGX0524D(GX24) | Dog | Guangxi | 2005 | HM486366 | **+** |
|  | CSH0330D(SH30) | Dog | Shanghai | 2003 | HM486400 | **+** |
|  | CSH0413D(SH13) | Dog | Shanghai | 2004 | HM486391 | **+** |
|  | CZJ0804D(D04) | Dog | Zhejiang | 2008 | HM486373 | **+** |
|  | CSH0323D(SH23) | Dog | Shanghai | 2003 | HM486357 | **+** |
|  | CAH0508D(AH8) | Dog | Anhui | 2005 | HM486361 | **+** |
|  | CSH0329D(SH29) | Dog | Shanghai | 2003 | HM486399 | **+** |
|  | CGX0609D(GX9) | Dog | Guangxi | 2006 | HM486365 | **+** |
|  | CGZ0517D(GZ17) | Dog | Guizhou | 2005 | HM486371 | **+** |
|  | CSD0708D(SD8) | Dog | Shandong | 2007 | HM486379 | **+** |
|  | CGZ0504D(GZ4) | Dog | Guizhou | 2005 | HM486367 | **+** |
|  | CSH0322D(SH22) | Dog | Shanghai | 2003 | HM486395 | **+** |
| **IIC** | F04 | Ferret badger | Zhejiang | 2008 | FJ712196 |  |
|  | F02 | Ferret badger | Zhejiang | 2008 | FJ712195 |  |
|  | ZJ-LA | Ferret badger | Zhejiang | 2008 | FJ598135 |  |
|  | JX08-48 | Ferret badger | Jiangxi | 2008 | FJ719753 |  |
|  | JX08-58 | Ferret badger | Jiangxi | 2008 | FJ719755 |  |
|  | XSN5 | Mouse | Zhejiang | 2008 | - | **+** |
|  | JX08-47 | Ferret badger | Jiangxi | 2008 | FJ719751 |  |
| **IID** | GX014 | Dog | Guangxi | 2003 | DQ866106 |  |
|  | GXHX | Dog | Guangxi | 2005 | DQ866119 |  |
|  | GX09 | Dog | Guangxi | 2003 | DQ866109 |  |
|  | GX08 | Dog | Guangxi | 2003 | DQ866108 |  |
|  | CGX0625D(GX25) | Dog | Guangxi | 2006 | EF990598 |  |
|  | CGZ0512D(GZ12) | Dog | Guizhou | 2005 | EF990571 |  |
|  | Guizhou_A101 | Dog | Guizhou | 2004 | DQ666289 |  |
|  | Guizhou_A103 | Dog | Guizhou | 2004 | DQ666290 |  |
|  | GX260 | Dog | Guangxi | 2004 | DQ866114 |  |
|  | GX195 | Dog | Guangxi | 2004 | DQ866112 |  |
|  | CGX0523D(GX23) | Dog | Guangxi | 2005 | EF990597 |  |
|  | CGX0614D(GX14) | Dog | Guangxi | 2006 | EF990589 |  |
|  | CGZ0516D(GZ16) | Dog | Guizhou | 2005 | EF990575 |  |
|  | CGX0615D(GX15) | Dog | Guangxi | 2006 | EF990590 |  |
|  | CGZ0506D(GZ6) | Dog | Guizhou | 2005 | EF990568 |  |
|  | CGZ0508D(GZ8) | Dog | Guizhou | 2005 | HM486368 | + |
|  | CGZ0514D(GZ14) | Dog | Guizhou | 2005 | EF990573 |  |
|  | FJ012 | Dog | Fujian | 2007 | FJ866829 |  |
|  | FJ013 | Dog | Fujian | 2007 | FJ866830 |  |
|  | FJ003 | Dog | Fujian | 2008 | FJ561728 |  |
|  | FJ014 | Dog | Fujian | 2007 | FJ866831 |  |
|  | FJ001 | Dog | Fujian | 2008 | FJ561726 |  |
|  | CSD0709D(SD9) | Dog | Shandong | 2007 | HM486381 | **+** |
|  | CHN0635H(HN35) | Human | Hunan | 2006 | EF990621 |  |
|  | FJ002 | Dog | Fujian | 2008 | FJ561727 |  |
|  | CGX0511D(GX11) | Dog | Guangxi | 2005 | EF990586 |  |
|  | GXSL | Dog | Guangxi | 2005 | DQ866120 |  |
|  | GXLA | Dog | Guangxi | 2003 | DQ866116 |  |
|  | GX091 | Dog | Guangxi | 2004 | DQ866110 |  |
|  | CGX0521D(GX21) | Dog | Guangxi | 2005 | EF990595 |  |
|  | CGX0601D(GX1) | Dog | Guangxi | 2006 | EF990578 |  |
|  | CGX0603D(GX3) | Dog | Guangxi | 2006 | EF990580 |  |
|  | CHN0633D(HN33) | Dog | Hunan | 2006 | EF990619 |  |
|  | CHN0610H(HN10) | Human | Hunan | 2006 | EF990620 |  |
|  | GX01 | Dog | Guangxi | 2004 | DQ866105 |  |
|  | CHN0642D(07HN42) | Dog | Hunan | 2007 | EF990622 |  |
|  |  |  |  |  |  |  |
| **Clade III** |  |  |  |  |  |  |
| **IIIA** | FJ006 | Dog | Fujian | 2008 | FJ561731 |  |
|  | FJ007 | Dog | Fujian | 2008 | FJ561732 |  |
|  | FJ004 | Dog | Fujian | 2008 | FJ561729 |  |
|  | FJ005 | Dog | Fujian | 2008 | FJ561730 |  |
|  | DRV | Deer | Jilin | - | DQ875051 |  |
|  | Henan_Sq48 | Dog | Henan | 2004 | DQ666305 |  |
|  | Hunan_Wg22 | Dog | Hunan | 2004 | DQ666310 |  |
|  | Hunan_Wg68 | Dog | Hunan | 2004 | DQ666313 |  |
|  | Guizhou_A173 | Dog | Guizhou | 2004 | DQ666293 |  |
|  | Henan_Sq17 | Dog | Henan | 2004 | DQ666301 |  |
|  | Jiangsu_Wx1 | Dog | Jiangsu | 2004 | DQ666321 |  |
|  | Guizhou_A10 | Human | Guizhou | 2004 | DQ666288 |  |
|  | Henan_Sq30 | Dog | Henan | 2004 | DQ666303 |  |
|  | Guizhou_A158 | Dog | Guizhou | 2004 | DQ666292 |  |
| **IIIB** | CGZ0501D(GZ1) | Dog | Guizhou | 2005 | EF990564 |  |
|  | CGZ0513D(GZ13) | Dog | Guizhou | 2005 | EF990572 |  |
|  | CGZ0505D(GZ5) | Dog | Guizhou | 2005 | EF990567 |  |
|  | CGZ0515D(GZ15) | Dog | Guizhou | 2005 | EF990574 |  |
|  | CGZ0510D(GZ10) | Dog | Guizhou | 2005 | EF990570 |  |
|  | CGZ0518H(GZ18) | Human | Guizhou | 2005 | EF990576 |  |
|  | CGX0617D(GX17) | Dog | Guangxi | 2006 | EF990591 |  |
|  | CHVC06 | Mouse |  | 2006 | EU282381 |  |
|  | MRV | Mouse | Henan | - | DQ875050 |  |
|  | Jiangsu_Yc63 | Dog | Jiangsu | 2004 | DQ666322 |  |
|  |  |  |  |  |  |  |
| **Clade IV** | MN1025B | Raccoon dog | Jilin | 2007 | EU652445 |  |
|  | NeiMeng925 | Raccoon dog | Henan | 2008 | FJ415313 |  |
|  | ABL | bat | Australia | 1996 | AF006497 |  |
