# Supplementary material for: The Spatial and Temporal Dynamics of Rabies in China
Source: PLoS Negl Trop Dis. 2012 May 1;6(5):e1640. doi: 10.1371/journal.pntd.0001640 (PMC3341336; doi:10.1371/journal.pntd.0001640)
Supplement: Table S2 — Details of MigraPhyla analysis to detect significant translocation events for (a) clade I and (b) clade 2 amongst the Chinese provinces from which samples were collected in this study. Top table shows number of translocation events predicted between pairs of provinces, lower table show statistical support for the events with P<0.05. (DOC) [file pntd.0001640.s002.doc]

**Table S2**

**Migration events in Clade I .**

Number of migration events

| From/to | HN | GX | GZ | YN | SH | AH | ZJ | JS | SD | FJ | HE |
| --- | --- | --- | --- | --- | --- | --- | --- | --- | --- | --- | --- |
| HN | 0 | 7 | 2 | 1 | 1 | 0 | 0 | 2 | 0 | 0 | 0 |
| GX | 0 | 0 | 1 | 0 | 0 | 0 | 0 | 0 | 0 | 0 | 0 |
| GZ | 0 | 1 | 0 | 1 | 0 | 0 | 0 | 0 | 0 | 0 | 0 |
| YN | 0 | 0 | 0 | 0 | 0 | 0 | 0 | 0 | 0 | 0 | 0 |
| SH | 0 | 0 | 0 | 0 | 0 | 0 | 0 | 0 | 0 | 0 | 0 |
| AH | 0 | 0 | 0 | 0 | 0 | 0 | 0 | 0 | 0 | 0 | 0 |
| ZJ | 0 | 0 | 0 | 0 | 0 | 0 | 0 | 0 | 0 | 0 | 0 |
| JS | 0 | 0 | 0 | 1 | 1 | 1 | 1 | 0 | 6 | 4 | 0 |
| SD | 0 | 0 | 0 | 0 | 0 | 0 | 0 | 0 | 0 | 0 | 0 |
| FJ | 0 | 0 | 0 | 0 | 0 | 0 | 0 | 0 | 0 | 0 | 0 |
| HE | 1 | 0 | 0 | 1 | 0 | 0 | 0 | 0 | 0 | 0 | 0 |

DELTRAN parsimony Monte Carlo p values

| From/to | HN | GX | GZ | YN | SH | AH | ZJ | JS | SD | FJ | HE |
| --- | --- | --- | --- | --- | --- | --- | --- | --- | --- | --- | --- |
| HN | 1 | 0.9981 | 1 | 0.9979 | 0.9999 | 1 | 1 | 0.9999 | 1 | 1 |  |
| GX | 1 | 1 | 0.2444 | 1 | 1 | 1 | 1 | 1 | 1 | 1 |  |
| GZ | 1 | 0.0459 | 1 | 0.0143 | 1 | 1 | 1 | 1 | 1 | 1 |  |
| YN | 1 | 1 | 1 | 1 | 1 | 1 | 1 | 1 | 1 | 1 |  |
| SH | 1 | 1 | 1 | 1 | 1 | 1 | 1 | 1 | 1 | 1 |  |
| AH | 1 | 1 | 1 | 1 | 1 | 1 | 1 | 1 | 1 | 1 |  |
| ZJ | 1 | 1 | 1 | 1 | 1 | 1 | 1 | 1 | 1 | 1 |  |
| JS | 1 | 1 | 1 | 0.0162 | 0.0304 | 0.0064 | 0.0321 | 1 | 0 | 0 |  |
| SD | 1 | 1 | 1 | 1 | 1 | 1 | 1 | 1 | 1 | 1 |  |
| FJ | 1 | 1 | 1 | 1 | 1 | 1 | 1 | 1 | 1 | 1 |  |
| HE | 0.0347 | 1 | 1 | 0.0118 | 1 | 1 | 1 | 1 | 1 | 1 | 1 |

HN:Hunan, GX:Guangxi, GZ:Guizhou, YN:Yunan, SH: Shanghai，ZJ:Zhejiang, JS:Jiangsu, SD:Shandong; FJ:Fujian, JX: Jiangxi，HE:Henan,

**Migration events in Clade II .**

number of migration events

| From/to | HN | GX | GZ | SH | AH | ZJ | JS | SD | FJ | JX |
| --- | --- | --- | --- | --- | --- | --- | --- | --- | --- | --- |
| HN | 0 | 2 | 1 | 1 | 0 | 1 | 0 | 0 | 0 | 1 |
| GX | 0 | 0 | 4 | 0 | 0 | 0 | 0 | 0 | 1 | 0 |
| GZ | 0 | 2 | 0 | 0 | 0 | 0 | 0 | 0 | 0 | 0 |
| SH | 0 | 3 | 4 | 0 | 2 | 3 | 1 | 2 | 0 | 0 |
| AH | 0 | 0 | 0 | 0 | 0 | 0 | 0 | 0 | 0 | 0 |
| ZJ | 0 | 0 | 0 | 0 | 0 | 0 | 0 | 0 | 0 | 0 |
| JS | 0 | 0 | 0 | 0 | 0 | 0 | 0 | 0 | 0 | 0 |
| SD | 0 | 0 | 0 | 0 | 0 | 0 | 0 | 0 | 0 | 0 |
| FJ | 1 | 0 | 0 | 0 | 0 | 0 | 0 | 1 | 0 | 0 |
| JX | 0 | 0 | 0 | 0 | 0 | 1 | 0 | 0 | 0 | 0 |

HN:Hunan, GX:Guangxi, GZ:Guizhou, YN:Yunan, SH: Shanghai，ZJ:Zhejiang, JS:Jiangsu, SD:Shandong; FJ:Fujian, JX: Jiangxi

DELTRAN parsimony Monte Carlo p values

| From/to | HN | GX | GZ | SH | AH | ZJ | JS | SD | FJ | JX |
| --- | --- | --- | --- | --- | --- | --- | --- | --- | --- | --- |
| HN | 1 | 0.0089 | 0.0247 | 0.0417 | 1 | 0.017 | 1 | 1 | 1 | 0.008 |
| GX | 1 | 1 | 0.9111 | 1 | 1 | 1 | 1 | 1 | 0.9834 | 1 |
| GZ | 1 | 0.1046 | 1 | 1 | 1 | 1 | 1 | 1 | 1 | 1 |
| SH | 1 | 0.413 | 0.2496 | 1 | 0.0836 | 0.2825 | 0.2079 | 0.1696 | 1 | 1 |
| AH | 1 | 1 | 1 | 1 | 1 | 1 | 1 | 1 | 1 | 1 |
| ZJ | 1 | 1 | 1 | 1 | 1 | 1 | 1 | 1 | 1 | 1 |
| JS | 1 | 1 | 1 | 1 | 1 | 1 | 1 | 1 | 1 | 1 |
| SD | 1 | 1 | 1 | 1 | 1 | 1 | 1 | 1 | 1 | 1 |
| FJ | 0.0177 | 1 | 1 | 1 | 1 | 1 | 1 | 0.0165 | 1 | 1 |
| JX | 1 | 1 | 1 | 1 | 1 | 0.0039 | 1 | 1 | 1 | 1 |
