# Supplementary material for: The Spatial and Temporal Dynamics of Rabies in China
Source: PLoS Negl Trop Dis. 2012 May 1;6(5):e1640. doi: 10.1371/journal.pntd.0001640 (PMC3341336; doi:10.1371/journal.pntd.0001640)
Supplement: Table S3 — Pairwise Mantel test results for correspondence using Spearman correlation ranks amongst six different distance matrices for (a) clade I and (b) clade II. noSamples: number of samples in the test set, geoDists: geographic distances between the central point of the provinces, UniFracPC1: distances between the provinces calculated from the first principal component, Migration: Number of migration events between pairs of provinces, NRI: Net Relatedness Index, NTI: Nearest Taxon Index. (DOC) [file pntd.0001640.s003.doc]

Table S3. Pairwise Mantel Test results across distance matrices:

Clade 1

|  | noSamples | geoDists | UniFracPC1 | Migration | NRI | NTI |
| --- | --- | --- | --- | --- | --- | --- |
| noSamples | 1 | 0.417 | 0.048 | 0.189 | 0.326 | 0.356 |
| geoDists | -0.031 | 1 | 0.013 | 0.984 | 0.045 | 0.045 |
| UniFracPC1 | 0.276 | 0.364 | 1 | 0.996 | 0.047 | 0.046 |
| Migration | 0.147 | -0.245 | -0.244 | 1 | 0.775 | 0.823 |
| NRI | 0.039 | 0.299 | 0.24 | -0.106 | 1 | 0.001 |
| NTI | 0.017 | 0.282 | 0.24 | -0.121 | 0.998 | 1 |

Clade II

|  | noSamples | geoDists | UniFracPC1 | Migration | NRI | NTI |
| --- | --- | --- | --- | --- | --- | --- |
| noSamples | 1 | 0.118 | 0.42 | 0.244 | 0.102 | 0.14 |
| geoDists | 0.216 | 1 | 0.374 | 0.703 | 0.542 | 0.515 |
| UniFracPC2 | -0.005 | 0.02 | 1 | 0.853 | 0.393 | 0.436 |
| Migration | 0.149 | -0.107 | -0.216 | 1 | 0.01 | 0.012 |
| NRI | 0.346 | -0.067 | -0.021 | 0.438 | 1 | 0.001 |
| NTI | 0.353 | -0.065 | -0.025 | 0.444 | 0.999 | 1 |

* Lower triangle -- Correlation coefficients of paired distance matrices

* Upper triangle -- P values (Empirical significance level from permutations)

**Supplementary Table 2.** Pairwise Mantel test results for correspondence test using Spearman correlation ranks amongst six different distance matrices for (a) clade I and (b) clade II. *noSamples*: number of samples in the test set, *geoDists*: geographic distances between the central point of the provinces, *UniFracPC1*: distances between the provinces calculated from the first principal component, *Migration*: Number of migration events between pairs of provinces, *NRI*: Net Relatedness Index, *NTI*: Nearest Taxon Index.
