# Supplementary material for: The Spatial and Temporal Dynamics of Rabies in China
Source: PLoS Negl Trop Dis. 2012 May 1;6(5):e1640. doi: 10.1371/journal.pntd.0001640 (PMC3341336; doi:10.1371/journal.pntd.0001640)
Supplement: Table S4 — Details of analysis of geographical composition of Clade I and Clade II. (DOC) [file pntd.0001640.s004.doc]

**Table S4**

| **CLADE-I** |  |  | **CLADE-II** |  |  |
| --- | --- | --- | --- | --- | --- |
|  | **SW** | **E** |  | **SW** | **E** |
| **2003** | 3 | 0 | **2003** | 4 | 9 |
| **2004** | 25 | 3 | **2004** | 9 | 6 |
| **2005** | 24 | 8 | **2005** | 14 | 2 |
| **2006** | 18 | 6 | **2006** | 10 | 3 |
| **2007** | 5 | 2 | **2007** | 1 | 5 |
| **2008** | 3 | 16 | **2008** | 0 | 14 |

**Method: contingency table—Chi-square test**

**Table s4.2**

**Clade 1**

H_0_: The ratio SW/E is independent of time period

H_a_: The ratio SW/E is associated with time period

|  | SW | E |
| --- | --- | --- |
| 2003-2005 | 52 | 11 |
| 2006-2008 | 26 | 24 |

Pearson's Chi-squared test with Yates' continuity correction

data: clade1[, 2:3]

X-squared = 10.7741, df = 1, p-value = 0.001029—extremely significant

We reject H_0_ at level of 0.05, namely, accept H_a_: The ratio SW/E is associated with time period

**Table s4.2**

**Clade2**

H_0_: The ratio SW/E is independent of time period

H_a_: The ratio SW/E is associated with time period

|  | SW | E |
| --- | --- | --- |
| 2003-2005 | 27 | 17 |
| 2006-2008 | 11 | 22 |

Pearson's Chi-squared test with Yates' continuity correction

data: clade2[, 2:3]

X-squared = 4.859, df = 1, p-value = 0.0275--significant

We reject H_0_ at level of 0.05, namely, accept H_a_: The ratio SW/E is associated with time period

**Table s4.3**

**2003-2005**

H_0_: The ratio SW/E is independent of clade

H_a_: The ratio SW/E is associated with clade

|  | clade1 | clade2 |
| --- | --- | --- |
| SW | 52 | 27 |
| E | 11 | 17 |

Pearson's Chi-squared test with Yates' continuity correction

data: y03[, 2:3]

X-squared = 4.9668, df = 1, p-value = 0.02584--significant

We reject H_0_ at level of 0.05, namely, accept H_a_: The ratio SW/E is associated with clade

**Table s4.4**

**2006-2008**

H_0_: The ratio SW/E is independent of clade

H_a_: The ratio SW/E is associated with clade

|  | clade1 | clade2 |
| --- | --- | --- |
| SW | 26 | 11 |
| E | 24 | 22 |

Pearson's Chi-squared test with Yates' continuity correction

data: y05[, 2:3]

X-squared = 2.0991, df = 1, p-value = 0.1474—not significant

We accept H_0_ at level of 0.05: The ratio SW/E is independent of clade
